# Non HIV-Associated Buffalo Hump as a Clinical Marker of Metabolic Disease

**DOI:** 10.3390/jcm14175997

**Published:** 2025-08-25

**Authors:** Nae-Ho Lee, Beom Jin Lim, Jin Yong Shin, Yoon Kyu Chung, Si-Gyun Roh

**Affiliations:** 1 Department of Plastic and Reconstructive Surgery, College of Medicine, Jeonbuk National University, Jeonju 54907, Republic of Korea; leenaeho@jbnu.ac.kr (N.-H.L.); dlaqjawls603@gmail.com (B.J.L.); psjyshin@gmail.com (J.Y.S.); ykchung@yonsei.ac.kr (Y.K.C.); 2 Research Institute of Clinical Medicine of Jeonbuk National University-Biomedical Research Institute of Jeonbuk National University Hospital, Jeonju 54907, Republic of Korea

**Keywords:** lipodystrophy, metabolic syndrome, adipose tissue, HIV protease inhibitors

## Abstract

**Background/Objectives**: Cervicodorsal lipodystrophy, commonly referred to as “buffalo hump,” has traditionally been associated with Human Immunodeficiency Virus (HIV)-related antiretroviral therapy. However, similar deformities may also occur independently of HIV treatment. This study aimed to investigate non HIV-associated buffalo hump as a potential clinical marker of underlying metabolic or endocrine disorders. **Methods**: We retrospectively reviewed 12 HIV-negative patients who presented with cervicodorsal lipodystrophy between 2012 and 2022. Patient demographics, laboratory values, and imaging findings were analyzed. All patients underwent surgical resection of a hypertrophic fat pad. Exploratory statistical analyses were performed using Mann–Whitney U and Fisher’s exact tests and Spearman’s correlation analysis. **Results**: These 12 patients had a mean age of 56.92 ± 16.69 years and a mean Body Mass Index (BMI) of 30.15 ± 4.59 kg/m^2^. Hypertension and diabetes were each present in 66.7% of patients, and hyperlipidemia in 75%. Three patients were newly diagnosed with metabolic disease. No significant differences were found between newly diagnosed and previously diagnosed patients in age (45.67 ± 21.46 vs. 60.67 ± 14.31 years, *p* = 0.194) or BMI (32.44 ± 2.39 vs. 29.39 ± 4.99 kg/m^2^, *p* = 0.145). Group differences in hypertension, diabetes, hyperlipidemia, or liver dysfunction were also not significant (all *p* > 0.49). No correlation was observed between age and BMI (ρ = −0.158, *p* = 0.624). **Conclusions**: Although the small sample size precludes definitive conclusions, the prevalence of obesity, hypertension, and diabetes in this cohort was notably higher than reported in Korean population-based surveys. These findings suggest that non HIV-associated buffalo hump may serve as an externally visible marker of systemic metabolic burden. Metabolic screening should be considered even in the absence of overt systemic disease.

## 1. Introduction

Buffalo hump, or cervicodorsal lipodystrophy, is a characteristic physical finding commonly recognized in Human Immunodeficiency Virus (HIV) patients receiving protease inhibitor-based antiretroviral therapy [1]. This condition, characterized by abnormal fat accumulation in the upper back and neck, has been widely documented as part of HIV-associated lipodystrophy syndrome [2,3]. Protease inhibitors, in particular, have been implicated in disrupting adipocyte differentiation and lipid metabolism, leading to fat redistribution [4,5,6]. However, in Korea, reverse transcriptase inhibitors are more commonly used, and the incidence of protease inhibitor-associated lipodystrophy is relatively low [7].

While traditionally associated with HIV treatment, buffalo hump may in fact represent a visible clinical clue to systemic disease in patients without HIV as well [8]. The presence of this fat accumulation in non-HIV patients often leads to an initial assumption of benign or aesthetic origin, yet it may reveal underlying pathologies that are otherwise asymptomatic. Few studies have focused on the clinical significance of cervicodorsal lipodystrophy in the absence of HIV, and even fewer have examined it within the Korean population [9].

Previous reports have described cervicodorsal fat accumulation in non-HIV contexts, including obesity, prolonged glucocorticoid exposure, and endocrine disorders such as Cushing syndrome, indicating that this phenotype is not exclusive to antiretroviral therapy. Moreover, simple anthropometric markers related to upper-body adiposity (e.g., neck circumference) have been linked to metabolic risk in non-HIV populations, underscoring the potential systemic relevance of this regional fat distribution [10].

Despite a strong historical association with HIV, cervicodorsal lipodystrophy is not exclusive to HIV-positive patients. It is also observed in conditions such as Cushing syndrome, long-term corticosteroid use, and obesity [11,12]. This raises the question: could buffalo hump in non-HIV patients serve as an early marker for metabolic or endocrine dysfunction? Given its visibility and the ease of clinical detection, this deformity may provide a valuable window into the patient’s broader health status.

In this study, we investigated the clinical features and systemic health profiles of non-HIV patients who presented with cervicodorsal lipodystrophy. We hypothesized that buffalo hump in these patients is not merely a cosmetic issue, but may reflect deeper, undiagnosed systemic disorders [13]. Our goal was to underscore the diagnostic value of this physical sign and promote a more comprehensive clinical approach to affected patients. To our knowledge, this is the first clinical review in Korea examining non HIV-associated cervicodorsal lipodystrophy as a visible marker of systemic disease.

## 2. Materials and Methods

We performed a retrospective chart review of patients diagnosed with cervicodorsal lipodystrophy at a single tertiary institution between January 2012 and December 2022. Inclusion criteria were the presence of a prominent fat pad in the cervicodorsal region and no prior diagnosis of HIV or exposure to antiretroviral therapy. Patient demographics, BMI, comorbidities, and laboratory data were reviewed. Laboratory evaluation included a standardized baseline panel consisting of fasting glucose, HbA1c, lipid profile, and liver function tests for all patients. Additional investigations, such as abdominal ultrasounds or endocrine hormone testing, were selectively performed when baseline results were abnormal. Although this was a retrospective study and a formal endocrine workup was not systematically performed for all patients, medical records were reviewed to exclude other common causes of cervicodorsal fat accumulation. None of these patients had a history of long-term corticosteroid use or exhibited typical clinical features of Cushing syndrome (e.g., moon face, wide striae, or proximal muscle weakness). In cases where endocrinology referral was made, further hormonal assessment (cortisol and ACTH testing) did not support Cushing syndrome.

Neck Magnetic Resonance Imaging (MRI) was performed for all patients. In addition to confirming diffuse adipose hypertrophy, MRI was valuable for excluding alternative pathologies such as lipoma, liposarcoma, or other soft tissue tumors. The imaging also delineated the depth and extent of adipose infiltration, which informed surgical planning. However, MRI findings themselves did not demonstrate predictive value regarding metabolic comorbidities. All patients underwent surgical excision of the fat pad under general anesthesia. Histologic examination confirmed mature adipose tissue without neoplastic changes. Postoperative outcomes and newly diagnosed systemic conditions were also recorded.

To minimize selection bias, all consecutive patients meeting the inclusion criteria during the study period were included. However, as a retrospective study, potential biases inherent to medical record review were acknowledged.

Descriptive and exploratory statistical analyses were performed using SPSS (v27.0, IBM Corp.). For readability, parametric measures of central tendency (means ± SD) were reported descriptively, while all inferential comparisons were conducted exclusively with nonparametric tests (Mann–Whitney U and Fisher’s exact tests). Correlations between variables were analyzed using Spearman’s rank correlation coefficient. Due to the limited sample size, all *p*-values should be interpreted with caution, and the findings regarded as exploratory.

At present, there is no standardized international algorithm or consensus guideline for the diagnosis of buffalo hump. In this study, diagnosis was based on visible cervicodorsal fat accumulation, further confirmed by MRI to exclude lipoma or other soft tissue masses, in accordance with previously published approaches.

## 3. Results

A total of 12 patients met the inclusion criteria. The mean age was 56.92 ± 16.69 years (range, 21 to 71), and the majority were female (10/12). None had a history of HIV or had received protease inhibitors or other antiretroviral medications.

**Figure 1:**
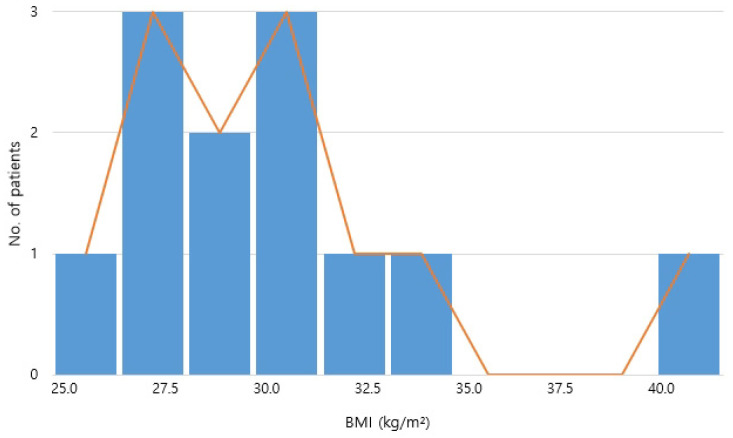
BMI distribution of all patients.

Eight patients had a prior diagnosis of hypertension, six had diabetes mellitus, and nine had elevated lipid profiles. Abnormal liver function test results were present in eight patients. Ten patients were classified as obese based on the World Health Organization (WHO) Asia–Pacific Body Mass Index (BMI) criteria, with a mean BMI of 30.15 ± 4.59 kg/m^2^ (Table 1). BMIs ranged from 25.0 to 40.2 kg/m^2^, with most patients falling within the overweight or obese range (Figure 1).

The above histogram shows the distribution of BMI values among all 12 patients with non HIV-associated cervicodorsal lipodystrophy. Most patients were in the obese range (BMI ≥ 30), emphasizing a potential relationship between the condition and metabolic burden.

Three patients were newly diagnosed with chronic metabolic disease following presentation for cervicodorsal lipodystrophy. All three had abnormal liver function tests and high BMIs, which prompted further laboratory and imaging evaluations. These findings suggest that the buffalo hump deformity served as the clinical entry point for uncovering underlying systemic disease.

### 3.1. Representative Case Presentations

#### 3.1.1. Case 1

**Figure 2:**
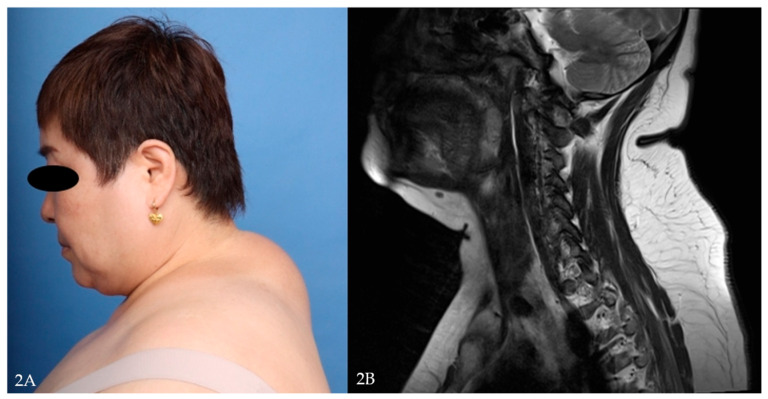
(**A**) Preoperative clinical photograph showing prominent buffalo hump. (**B**) Sagittal T2-weighted MRI demonstrating diffuse subcutaneous fat accumulation in the cervicodorsal region.

**Figure 3:**
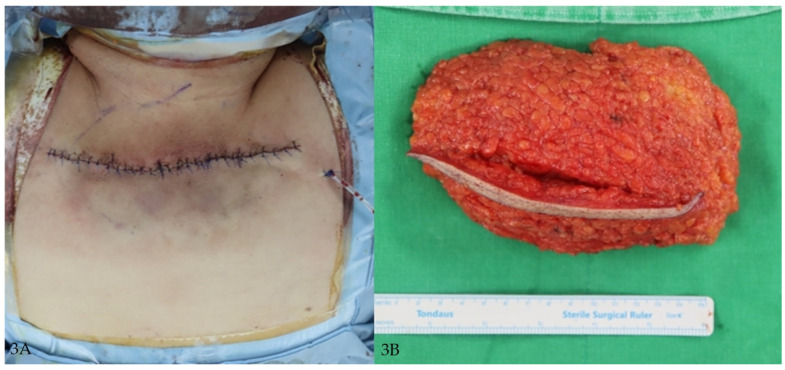
(**A**) Immediate postoperative photograph showing primary closure after excision. (**B**) Gross photograph of excised specimen consisting of adipose tissue with overlying fibrous septa.

A 60-year-old female with no prior chronic illness presented with progressive fat accumulation in the cervicodorsal region. Workup revealed elevated liver enzymes, a fasting glucose of 144 mg/dL, and total cholesterol of 271 mg/dL. She was subsequently diagnosed with type 2 diabetes and hyperlipidemia. Her BMI was 34.2 kg/m^2^. Surgical excision of the fat pad was performed without complications (Figure 2A,B and Figure 3A,B).

#### 3.1.2. Case 2

**Figure 4:**
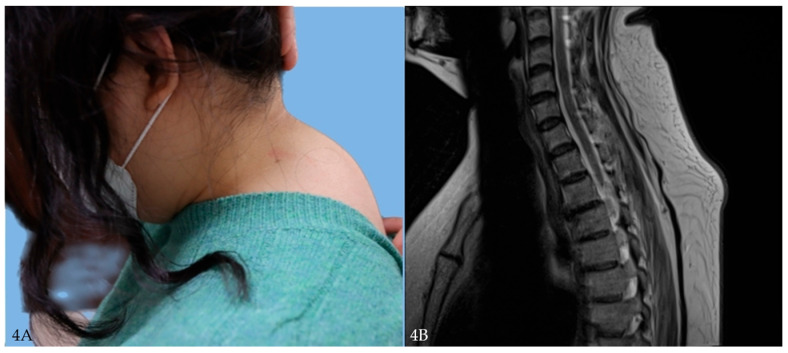
(**A**) Preoperative clinical photograph showing prominent buffalo hump. (**B**) Sagittal T2-weighted MRI demonstrating diffuse subcutaneous fat accumulation in the cervicodorsal region.

**Figure 5:**
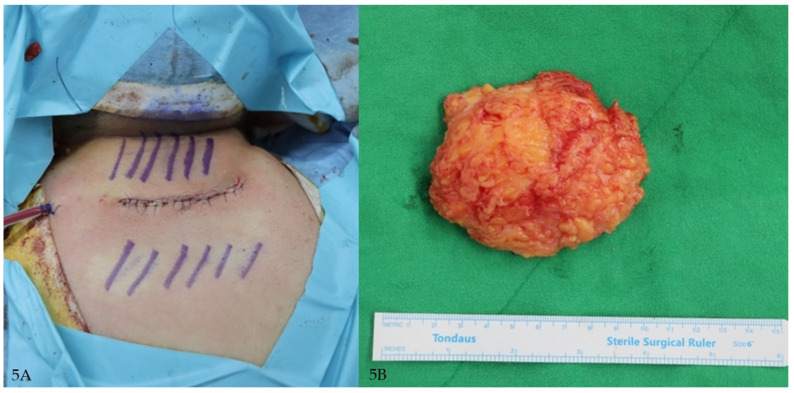
(**A**) Immediate postoperative clinical photograph showing primary closure after excision. (**B**) Gross photograph of excised specimen consisting of adipose tissue with overlying fibrous septa.

A 21-year-old woman with a visible buffalo hump underwent evaluation for cosmetic concerns. Laboratory tests revealed hypertriglyceridemia and included an abnormal liver function test (LFT). Her BMI was 33.5 kg/m^2^. She was referred to internal medicine and newly diagnosed with nonalcoholic fatty liver disease (NAFLD) (Figure 4A,B and Figure 5A,B).

#### 3.1.3. Case 3

**Figure 6:**
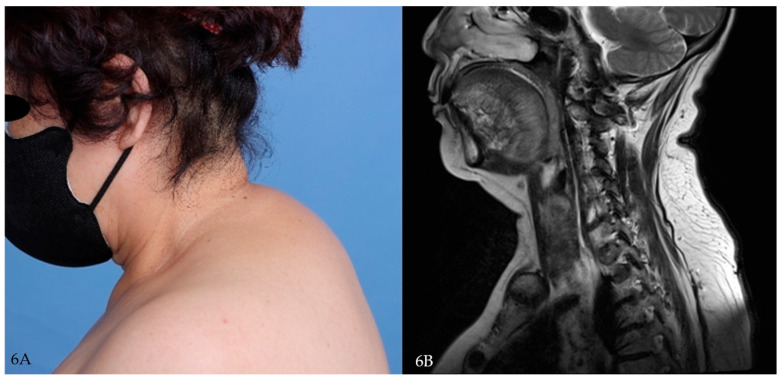
(**A**) Preoperative clinical photograph showing prominent buffalo hump. (**B**) Sagittal T2-weighted MRI demonstrating diffuse subcutaneous fat accumulation in the cervicodorsal region.

**Figure 7:**
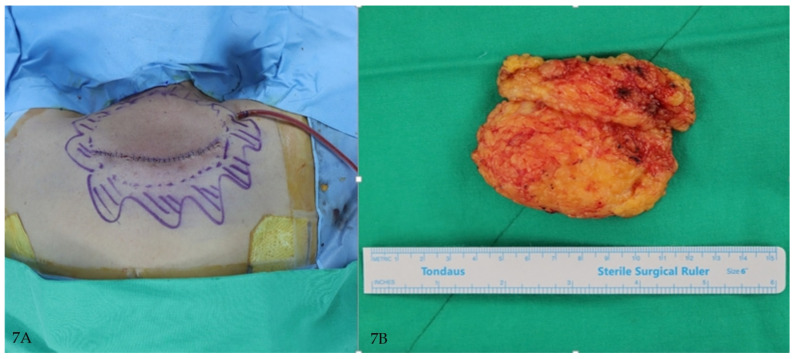
(**A**) Immediate postoperative clinical photograph showing primary closure after excision. (**B**) Gross photograph of excised specimen consisting of adipose tissue with overlying fibrous septa.

A 56-year-old male was referred for cervicodorsal lipodystrophy. He had no prior diagnoses but was found to have elevated HbA1c (7.1%) and abnormal liver enzymes. His BMI was 33.9 kg/m^2^, and he was subsequently diagnosed with type 2 diabetes and obesity-related liver dysfunction (Figure 6A,B and Figure 7A,B).

### 3.2. Statistical Findings

**Figure 8:**
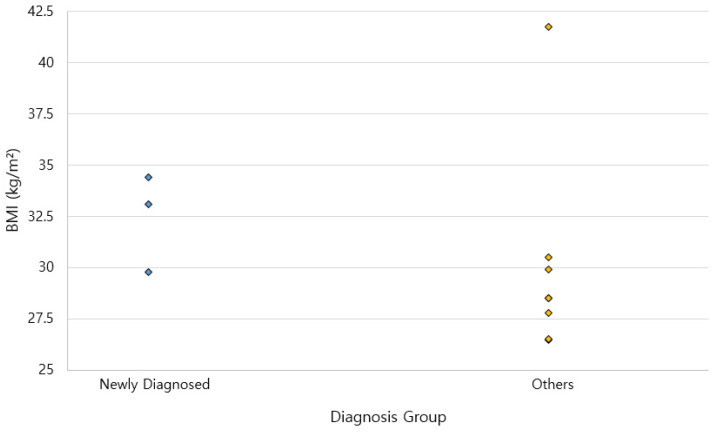
BMI comparison between groups. Blue dots represent newly diagnosed patients, red dots represent others.

No significant differences were found between the newly diagnosed group and others in age (45.67 ± 21.46 vs. 60.67 ± 14.31 years, *p* = 0.194) or BMI (32.44 ± 2.39 vs. 29.39 ± 4.99 kg/m^2^, *p* = 0.145). Similarly, group differences were not statistically significant for the prevalence of hypertension, diabetes mellitus, hyperlipidemia, and liver dysfunction (all *p* > 0.49). In addition, there was no significant correlation between age and BMI (Spearman’s ρ = −0.158, *p* = 0.624) (Table 2). Newly diagnosed patients had a slightly higher average BMI, but the difference was not statistically significant (Figure 8). These results, however, must be interpreted with caution given the small sample size and lack of statistical power. Non-significant results in this context are inconclusive rather than evidence of no association. Although not statistically significant, the observed patterns may still suggest that cervicodorsal lipodystrophy reflects systemic disease burden, especially in obese individuals.

A scatter plot compared individual BMI values between newly diagnosed patients and those with existing or no metabolic disease. While not statistically significant, the newly diagnosed group exhibited a higher mean BMI, suggesting a possible clinical trend.

## 4. Discussion

Cervicodorsal lipodystrophy, often referred to as “buffalo hump”, has been extensively described in the context of HIV-related antiretroviral therapy. However, its presence in patients without HIV infection has received relatively little attention [14]. Our study highlights the clinical relevance of non HIV-associated cervicodorsal lipodystrophy as a potential marker for systemic disease. In more than half of our patients, the deformity was associated with at least one metabolic comorbidity, and in several cases, it prompted the initial diagnosis of chronic conditions [15,16]. In three patients, buffalo hump led to the initial diagnosis of underlying conditions such as diabetes, hyperlipidemia, or fatty liver disease.

Although statistical significance was not achieved, the patterns observed in Table 2 suggest that newly diagnosed patients tended to have a slightly higher BMI (32.44 ± 2.39 kg/m^2^) compared to others (29.39 ± 4.99 kg/m^2^), and were younger (45.67 ± 21.46 vs. 60.67 ± 14.31 years, *p* = 0.194). These differences, while not statistically significant due to the small sample size, suggest a distinctive clinical profile. All three newly diagnosed patients exhibited abnormal liver function compared to 55.6% of others. Similarly, age and BMI showed no significant correlation (Spearman’s ρ = –0.158, *p* = 0.624). While these findings cannot confirm a direct association, they support the hypothesis that cervicodorsal lipodystrophy in obese individuals warrants investigation for underlying metabolic disorders.

Although a direct control group was unavailable in this retrospective series, a comparison with Korean population-based data revealed notable differences. In our cohort, the mean BMI among newly diagnosed patients was 32.44 ± 2.39 kg/m^2^, while the prevalence of hypertension and diabetes was 66.7% each. In contrast, national survey data report mean BMIs of 24.6 kg/m^2^ for men and 23.2 kg/m^2^ for women, with prevalence rates of about 30% for hypertension and 15.5% for diabetes in age-matched adults. These discrepancies reinforce the hypothesis that cervicodorsal lipodystrophy may serve as a visible clinical marker of systemic metabolic burden [17,18,19].

The pathophysiology of non HIV-associated buffalo hump is likely multifactorial. Obesity, insulin resistance, and dyslipidemia contribute to aberrant fat distribution patterns [20,21]. Similar to the effects seen in Cushing syndrome, prolonged exposure to metabolic stress can lead to regional adipose hypertrophy. Although our study did not systematically test for Cushing syndrome due to its retrospective design, the presence of multiple overlapping features—hypertension, diabetes, and central obesity—suggests that buffalo hump may be an outward manifestation of internal endocrine dysregulation [22].

Beyond these factors, previous studies have highlighted that chronic obesity-related adipose tissue hypertrophy, insulin resistance, and dyslipidemia may act synergistically to induce regional fat redistribution [23]. Subclinical cortisol excess has also been proposed as a contributor, although overt Cushing syndrome was not present in our cohort. These mechanisms parallel, but are not identical to, those described in HIV-associated lipodystrophy, suggesting a distinct but overlapping pathophysiologic process [24]. Future studies should investigate the role of biochemical screening for Cushing syndrome or other endocrine abnormalities in such patients.

From a clinical perspective, this finding has important implications. Patients often seek medical attention for cosmetic correction of cervicodorsal lipodystrophy, yet the deformity may be an early sign of more serious systemic illness. Surgeons and primary care providers should consider comprehensive metabolic screening in these cases rather than limiting evaluation to surgical correction alone [25,26]. Although BMI itself is not a diagnostic criterion for buffalo hump, the frequent coexistence of obesity and cervicodorsal fat accumulation in our cohort coincided with metabolic abnormalities in a majority of cases. This suggests that clinicians should be particularly attentive to systemic evaluation when buffalo hump presents in obese individuals.

Additionally, the surgical treatment of buffalo hump is often approached purely from an aesthetic perspective. However, our findings suggest that the deformity should be reframed as a potential diagnostic feature [27,28]. In several of our cases, the decision to perform surgical intervention created an opportunity to detect underlying metabolic abnormalities that had previously gone unnoticed. Therefore, a multidisciplinary approach involving endocrinology, internal medicine, and plastic surgery should be encouraged when managing these patients. We recommend that surgical evaluation of cervicodorsal lipodystrophy include baseline laboratory screening for metabolic or hepatic abnormalities, even in the absence of prior systemic diagnosis.

This visibility presents an important opportunity for early detection of metabolic dysfunction, especially in populations with limited access to health screening. In low-resource settings or in populations with limited access to regular health screening, such physical signs may play a critical role in prompting further evaluation.

Finally, this study contributes to the growing recognition that non HIV-associated lipodystrophy syndromes remain under-characterized in the medical literature. While HIV-related fat redistribution syndromes have been extensively studied, few investigations have addressed lipodystrophy in the general population without antiretroviral exposure. In addition, similar observations of cervicodorsal lipodystrophy in non-HIV populations have been reported in other countries, including in Europe and North America, often associated with obesity, long-term corticosteroid use, or endocrine abnormalities. These reports suggest that this phenomenon is not limited to the Korean population, although systematic data remain scarce [29,30]. Our findings call attention to this overlooked phenotype and support the need for larger prospective studies to define its prevalence, risk factors, and optimal management strategies.

In summary, although statistical associations were not observed in this study, the abnormalities of metabolic conditions in patients with cervicodorsal lipodystrophy warrant clinical attention. In conclusion, non HIV-associated buffalo hump should not be regarded solely as an HIV-related or cosmetic issue. Our findings are exploratory and hypothesis-generating, and the absence of statistically significant associations should be interpreted as inconclusive rather than evidence of no relationship. Larger, prospective studies with standardized measurements and BMI-matched controls are required to validate these preliminary observations. Nonetheless, cervicodorsal lipodystrophy remains a visible clinical sign that warrants further evaluation, and baseline metabolic screening should be considered in affected patients, particularly those with obesity, to facilitate early detection of systemic disease. Early identification and management of such comorbidities could improve long-term outcomes and reduce the risk of complications.

The limitations of this study include its retrospective nature, single-center scope, and small sample size. The absence of quantitative imaging or anthropometric measurements is another limitation. While all patients underwent MRI, volumetric analysis of the adipose deposit and neck circumference data were not consistently available due to the retrospective design. These parameters could potentially provide correlations with metabolic outcomes and should be incorporated in future prospective studies. Another limitation is the lack of a BMI-matched control group without cervicodorsal lipodystrophy. Such a comparison would strengthen the analysis, but was not feasible in this retrospective series, as patients without buffalo hump are rarely subjected to comparable MRI or surgical procedures. Accordingly, we reframed our analysis primarily as a descriptive case series with exploratory comparisons, supplemented by literature-based comparisons to population data. While our exploratory comparisons suggest clinical trends, further statistical validation in larger cohorts is needed to determine the strength of these associations.

## Figures and Tables

**Table 1:** Clinical characteristics of all patients.

	All Patients (*n* = 12)
Mean Age (years)	56.92 ± 16.69
Sex (Male:Female)	2:10
Mean BMI (kg/m^2^)	30.15 ± 4.59
Hypertension (%)	8 (66.7%)
Diabetes Mellitus (%)	6 (50%)
Abnormal LFT (%)	8 (66.7%)
Hyperlipidemia (%)	9 (75%)

Values are presented as mean ± standard deviation or number (%). BMI = body mass index; LFT = liver function test.

**Table 2:** Comparison between newly diagnosed patients and others.

Variables	Newly Diagnosed (*n* = 3)	Others (*n* = 9)	*p*-Value
Mean Age (years)	45.67 ± 21.46	60.67 ± 14.31	0.194
Female (%)	3 (100%)	7 (77.8%)	
Mean BMI (kg/m^2^)	32.44 ± 2.39	29.39 ± 4.99	0.145
Hypertension (%)	2 (66.7%)	6 (66.7%)	1.000
Diabetes Mellitus (%)	2 (66.7%)	4 (44.4%)	1.000
Abnormal LFT (%)	3 (100%)	5 (55.6%)	1.000
Hyperlipidemia (%)	3 (100%)	6 (66.7%)	0.509

Values are presented as mean ± standard deviation or number (%). *p*-values were calculated using the Mann–Whitney U test for continuous variables and Fisher’s exact test for categorical variables. *p* < 0.05 denotes statistical significance. BMI = body mass index; LFT = liver function test.

## Data Availability

Data presented in this study are available on request from the corresponding author.

## Abbreviations

The following abbreviations are used in this manuscript:

HIV	Human Immunodeficiency Virus
BMI	Body Mass Index
MRI	Magnetic Resonance Imaging
SPSS	Statistical Package for the Social Sciences
WHO	World Health Organization
LFT	Liver Function Test
NAFLD	Nonalcoholic Fatty Liver Disease
